# Sodium Houttuyfonate Inhibits Bleomycin Induced Pulmonary Fibrosis in Mice

**DOI:** 10.3389/fphar.2021.596492

**Published:** 2021-02-25

**Authors:** Yun-hui Shen, Ming-han Cheng, Xin-yu Liu, De-wei Zhu, Jian Gao

**Affiliations:** ^1^ School of Pharmacy, Shanghai University of Traditional Chinese Medicine, Shanghai, China; ^2^ The Second Hospital of Dalian Medical University, Dalian, China

**Keywords:** pulmonary fibrosis, sodium houttuyfonate, bleomycin, pulmonary function, transforming growth factor-β (TGF-β), Smad

## Abstract

Pulmonary fibrosis (PF) could severely disrupt the normal lung architecture and function with fatal consequences. Currently, there is no effective treatment for PF or idiopathic pulmonary fibrosis (IPF). The aim of this study was to investigate the effects of Sodium Houttuyfonate (SH) on bleomycin (BLM) induced PF mice model. Our results indicated that SH could attenuate BLM induced lung injury by reducing the inflammation, fibrogenesis and lung/body weight ratio. The proposed mechanisms for the protective effects of SH include: 1) improvement of pulmonary function in BLM mice, for instance, it can elevate the vital capacity (VC), increase the forced expiratory flow at 50% of forced vital capacity (FEF50) and improve other pulmonary function indices; 2) inhibition of collagen formation in BLM mice; 3) attenuation of the elevation of inflammatory cytokines, such as interleukin-1β (IL-1β), IL-6, and tumor necrosis factor-α (TNF-α), which are triggered by BLM administration; 4) reduction of the mRNA level and protein production of transforming growth factor-β1 (TGF-β1) in BLM mice. Furthermore, it was found that the protective effects of SH against BLM induced PF in mice was comparable to that of prednisone acetate (PA) tablets, a widely used drug for immunological diseases. Although *Houttuynia Cordata* Thunb has been widely used in China for lung infection and inflammation, the mechanism has not yet been fully elucidated. Our study provides the evidence that SH is an effective compound against pulmonary injury, irritation and fibrogenesis.

## Highlights

1. Sodium Houttuyfonate (SH) significantly ameliorates Pulmonary fibrosis (PF);
2. SH improved the lung function. These indexes include Cdyn, VC, and MMEF etc;
3. SH inhibited the expression of inflammatory cytokine IL-1β, IL-6, TNF-α and TGF-β1;
4. SH suppressed the pulmonary hydroxyproline and the collagen I/collagen III ratio;
5. SH reduced the lung inflammatory cell infiltration and collagen deposition by histological examinations.

## Introduction

Pulmonary fibrosis (PF), the progressive disorder characterized by the destruction of functional lung parenchyma and architectural remodeling of tissues, often causes fatal consequences. PF can be triggered by a persistent irritant and they may be known factors, such as toxic chemicals, allergens, radiation and environmental particles (Wilson and Wynn, 2009; Xu et al., 2019). Some unknown factors can also trigger idiopathic pulmonary fibrosis (IPF), which is one of the most common pulmonary fibrotic conditions, aberrant activation of alveolar epithelial cells and fibroblasts in an aging lung is the key to the pathogenesis of IPF (Selman and Pardo, 2014). This progressive and fatal disease is chronic and characterized by bilateral pulmonary interstitial infiltration, restriction on pulmonary function testing, and progressive dyspnea with respiratory failure (Degryse and Lawson, 2011; Hochhegger et al., 2019). Pulmonary wound-healing responses following lung damage include inflammation and repair, often accompanied by the rise of chemokines, cytokines and growth factors. Dozens of potential novel therapeutic targets were discovered (Kingwell, 2014; Staitieh et al., 2015), including specific cytokines, such as transforming growth factor-β (TGF-β), connective tissue growth factor (CTGF), interleukin-13 (IL-13) and interferon-γ(IFN-γ) (Scotton and Chambers, 2007).

Various PF animal models have been developed to search for potential pharmacological therapies against IPF. The bleomycin (BLM) model in rodents (mouse, rat and hamster) is most commonly used, which could cause a pulmonary pathological change similar to that of patients receiving chemotherapy (Moeller et al., 2008; Della et al., 2015). Mice of Kunming species, have been proved to be an applicable animal model for bleomycin-induced PF model (Cui et al., 2019; Xin et al., 2019). The frequent use of BLM in animal models of PF is due to the reason that inflammation and fibrosis are its major adverse effects occurring quickly. The ascending in pro-inflammatory cytokines (tumor necrosis factor-α(TNF-α), IFN-γ, IL-1, IL-6) is followed by a rise in pro-fibrotic markers (TGF-β, procollagen-1, fibronectin), peaking around day 14 (Chaudhary et al., 2006). Using the BLM model, several compounds have been tested in clinical trials, including etanercept, imatinib, prednisone, and TGF-β antibody (Walter et al., 2006).

Although great progress in fundamental and clinical study of IPF has been made, there is no reliable therapy (Gross and Hunninghake, 2001). It is necessary to find promising new drugs which are safe for IPF. Presently, the pharmacotherapy agent options are pirfenidone and nintedanib, which only received conditional suggestions in view of clinical effects, costs and safety profiles (Zhang et al., 2018a; Chen et al., 2018; Saito et al., 2019). Cortical hormone is also employed to treat PF, and prednisone acetate (PA) is the common selected drug for positive control studies in bleomycin-induced PF model (Ji et al., 2016; Tao et al., 2017; Zhang et al., 2018b; Chen et al., 2018). Traditional herbal medicines, with their efficacy and advantages have being investigated over years, have been considered to be potential candidates for treating IPF (Yao and Jiang, 2003). In pre-clinical studies, some single herbs or herbal extracts, such as Cordyceps sinesis, hedysarum polybotyssaccharide and costunolide (CN), have been reported to downregulate TGF-β, IL-4 *in vivo* or *in vitro* (Liu et al., 2019). Furthermore, some herbal medicine formulations are currently under clinical trials (Yang et al., 2009). Recently, more researches have been carried out to examine the mechanisms of herbs affecting BLM-induced PF. For instance, the quercetin improved PF through regulating the expression of fibrosis key factors such as fibronectin, Collagen I, Collagen III and hydroxyproline (Zhang et al., 2018a). Polysaccharides from Dendrobium officinale could significantly ameliorated pulmonary inflammation and fibrosis via inactivation of TGFβ1-Smad2/3 signaling pathway (Chen et al., 2018).

*Houttuynia Cordata* Thunb (family Saururaceae) is used in many herbal prescriptions for its antimicrobial and anti-inflammatory effects. Houttuynin is the key ingredient in the volatile oil from *Houttuynia Cordata* Thunb*.* Sodium Houttuyfonate (SH), an addition compound from sodium bisulfite and houttuynin, has been used as a novel therapy for bronchitis and upper respiratory infections for years in China (See Supplementary Figure S1 for structure). Previously, it has been found that SH could block myocardial fibrosis to slow down or inhibit the progression of ventrical remodeling and heart failure induced by pressure overload in rats, by inhibiting the initiation of the renin-angiotensin-aldosterone system and decreasing production of collagen and the excretion of TNF-α (Gao et al., 2010; Zheng et al., 2018). Therefore, SH plays a role in fibrosis of viscera. Yuan’s group also showed that SH could treat cationic Bovine Serum Albumin (C-BSA) induced membranous glomerulonephritis in BALB/c mice by inhibiting the elevation of NF-κB protein and translation of MCP-1 (Pan et al., 2010). Lin’s group in Taiwan reported that *Houttuynia cordata* Thunb has a protective effect against BLM-induced PF in rats; however, only the water extract of *Houttuynia cordata* Thunb was used for Lin’s research and the effective compounds and mechanisms of actions remained unclear (Ng et al., 2007). The present study aims to investigate the effects of SH on BLM-induced PF in mice and the related mechanisms.

## Materials and Methods

### Chemicals

Sodium Houttuyfonate (SH, Shanghai Qingping Pharmaceutical Company Ltd., China. Lot No. 100305, Purity 99.4%); Bleomycin hydrochloride (BLM, Haerbin Bolai Pharmaceutical Company Ltd. of Heilongjiang Province, China. Lot No. 100131), Prednisone acetate tablets (PA, Zhejiang Xianju Pharmaceutical Company Ltd. of Zhejiang Province, China. Lot No. 100341).

On the experimental days, SH was dissolved in normal saline at a concentration of 90 mg/kg and 45 mg/kg. PA was dissolved in normal saline at a concentration of 5 mg/kg.

### Animal Care and Handling

Kunming mice, being proved to be an applicable animal model for bleomycin-induced PF model (Cui et al., 2019; Xin et al., 2019), were obtained from Shanghai Experimental Animal Center, Chinese Academy of Sciences. The animals were housed in stainless steel cages and observed under a 12 h light-12 h dark cycle in a well-ventilated room at 23 ± 2°C. They were fed with standard pellet food and tap water *ad libitum*. Being approved by the Animal Care and Use Committee of Shanghai University of Traditional Chinese Medicine, the study was performed in accordance with the guidelines for care and handling of animals of the National Institute of Health.

### Bleomycin-Induced Mice Model

In the pre-experiment stage, two different dosages of BLM (2.5 mg/kg, 5 mg/kg) were given to 10 mice (5 mice for each group) in a small-scale preliminary experiment. On the 7th and 14th day, the mice were anesthetized with pentobarbital sodium (50 mg/kg) intraperitoneally. The lungs were collected for histological examination. The lung tissues of the mice in the 5 mg/kg treatment group showed fibrotic morphology. No significant pathological changes were observed in the 2.5 mg/kg group (Data not shown). Therefore, 5 mg/kg of BLM was used to generate the BLM induced PF mice model in subsequent experiments (Moeller et al., 2008; Mouratis and Aidinis, 2011; Cui et al., 2019; Xin et al., 2019).

### Administration of Reagents and Study Groups

Oral administration dose of SH in adults: 180mg–270 mg/day, so the equivalent dose for mice is 30–45 mg/kg. With reference to previous report and small-scale preliminary experiments, we employed two different administration concentrations (45 mg/kg and 90 mg/kg dosage). One hundred and Thirty mice (male, 8 weeks old, 18–22 g) were randomly divided into the following six groups, 120 mice for five groups: sham-operation control group (Control); BLM treatment group (BLM); PA treatment group (BLM + PA); high concentration SH treatment group (BLM + SH High); and low concentration SH treatment group (BLM + SH Low); 10 mice were treated with SH (90 mg/kg) alone (data shown in Supplementary Material). On the operation day, mice in the control group received 1 ml/kg 0.9% sterile saline intratracheally under pentobarbital sodium anesthesia, while mice in other groups received a single dose of BLM intratracheally (5 mg in 1 ml sterile saline/body weight (kg)). From the first day after modeling, the control and BLM groups received saline (orally, 10 ml/kg body weight) once a day. SH was administered orally once a day to both the high (90 mg/kg) and low (45 mg/kg) concentration SH groups. On Day 7, 14 and 28, Pentobarbital sodium (50 mg/kg) was injected intraperitoneally for 6, 8 and 10 mice in each group respectively. Briefly, the pulmonary function of mice was recorded through a computer-controlled Animal pulmonary function measurement system (PFT; Buxco) consisting of small-animal ventilator, signal amplifier, positive and negative pressure tank, plethysmograph and control switch. Following exsanguinations, the chest cavity was opened. The left lung was frozen in liquid nitrogen for determination of cytokines and hydroxyproline, the TGF-β1 and pSmad2/3 protein in the left lung tissue (about 100 mg) were analyzed by Western blot on the 28th day, while the right one was divided into two parts; half was frozen in liquid nitrogen for Q-PCR measurements and the remaining half was used for morphological observation.

### Pulmonary Function Test

After anesthesia, the mice were fixed on the board in the body tracing box by supine position, with tracheal tube inserting into trachea and connecting with the body tracing box. The change of lung volume was obtained indirectly by the change of gas pressure in the box. The data were analyzed and processed by Animal pulmonary function measurement system, including dynamic pulmonary compliance (Cdyn), resistance of lung (RL), vital capacity (VC) and forced expiratory flow at 50% of forced vital capacity (FEF50), forced expiratory flow at 75% of forced vital capacity (FEF75), maximum mid-expiratory flow (MMEF).

### Lung/Body Weight Ratio

At the seventh, 14th and 28th day of administration, mice in each treatment group were fasted with free access to water for 12 h. Mice were sacrificed after anesthesia and the body weight (BW) was recorded, then the whole lung was taken and weighed (pulmonary weight, PW), lung weight indexes were calculated by the equation PI = PW/BW*100.

### Histological Examination and Evaluation

The lungs were immersed in buffered formalin (10%) for 48 h, then embedded in paraffin and sliced (5 µm), staining with H and E, or Sirius red. Each sample slice was photographed (×200 magnification) under the microscope (Olympus B×51, 167 Japan). All photos were analyzed with the image-Pro Plus 6.3 analyzing software (Media Cybernetics, Bethesda, MD, United States) by computer. Type I and type III collagen accumulation in the interstitial space of the lung was assessed by polarized light microscopy. Type I or type III collagen volume fraction (CVF) was also determined with Image-Pro 6.3 analyzing software.

### ELISA

IL-1β, IL-6 and TNF-α in the lung were quantified by ELISA with mouse immunoassay kits (Xitang Biotechnology Corporation of Shanghai, China. lot No. 201007033, 201006281, 201007102). 10% supernatant of lung homogenates was added to the wells, which were coated with monoclonal antibody against IL-1β, IL-6 or TNF-α. After five washes, the biotinylated antibody against IL-1β, IL-6 or TNF-α, the peroxidase-conjugated avidin, and the chromogenic substrates were added to each well sequentially. The absorbance was read at 450 nm in an ELISA plate reader.

### RNA Isolation, Reverse Transcription, and Real-Time Quantitative PCR

Total RNA was isolated from each lung tissue sample using Trizol (Invitrogen, United States). Reverse transcription was performed using SuperScript II reverse transcriptase (Invitrogen). Quantitative real-time PCR (qRT-PCR) was carried out with a light cycle analyzer (Roche Diagnostics, Germany) using SYBR Green (Applied Biosystems, United States) to detect the products. The reactions were cycled 40 times under the appropriate parameters for the primers and the fluorescence was measured at the end of each cycle to construct the amplification curve. GAPDH was loaded as the control, with primers F: 5′- ACA​ACT​TTG​GCA​TTG​TGG​AA -3′ and R: 5′- GAT​GCA​GGG​ATG​ATG​TTC​TG -3′. Specific primers for mouse TGF-β1 were used, F: 5′- GGG​GCC​GGT​CTA​TGA​CGA​GC-3′ and R: 5′- TCT​GGG​CTT​CCA​TTT​CCA​CAT​CCG -3′. All determinations were performed at least twice.

### Western Blot Analysis of TGF-β1 and pSmad2/3 Proteins

Total proteins, which extracted from lung tissue, homogenized in tissue lysis buffer. The lysates were centrifuged at 12,000 rpm for 15 min at 4°C, and the supernatant was collected and stored at −80°C. The Coomassie Brilliant Blue was performed to quantitate protein concentration. 80 μg/well protein were electrophoresed using 10% sodium dodecyl sulphate (SDS) polyacrylamide gel (loading buffer, running buffer, transfer buffer, PIPA lysate, SDS-PAGE gel preparation kit, stripping buffer were purchased from Jiangsu Biyuntian Biotechnology Research Institute) for analysis of expressed TGF-β1 and the phosphorylated Smad2/3. After electrophoresis, the gel was wet transferred on to Polyvinylidene fluoride (pvdf) membrane. The membrane was blocked with milk (10%) for 90 min after eluted by Tris Buffered Saline Tween (TBST) four times and Ten minutes at a time, and then eluted again. Then incubated overnight at 4°C with primary antibodies: anti-TGF-β1 (abcam, United Kingdom), anti-pSmad2/3 (Cell Signaling Technology), and anti-GAPDH (Epitomics, United States). After primary antibody incubation and washing, incubated 2 h using secondary antibody. Blots were developed with visualized by Chemidoc imaging and GAPDH was used as a loading positive control.

### Hydroxyproline Assay

Lung hydroxyproline levels were determined spectrophotometrically by absorbance at 550 nm with the Hydroxyproline Kit (Nanjing Jiancheng Institute of Bioengineering, Nanjing, China) and the results were expressed as micrograms of hydroxyproline per milligram of lung tissue.

### Statistical Analysis

All values were expressed as mean ± standard deviation (SD). Statistical analysis was performed by one-way analysis of variance for multiple comparisons, Student’s t test was used if the variance between the two groups was homogeneous. Dunnet’s T3 test was used if the variance was not homogeneous. Mann Whitney test was used for the data of non-normal distribution. Values of *p* < 0.05 were considered statistically significant for all analyses.

## Results

### Effects of SH on Pulmonary Function in BLM Mice

**FIGURE 1:**
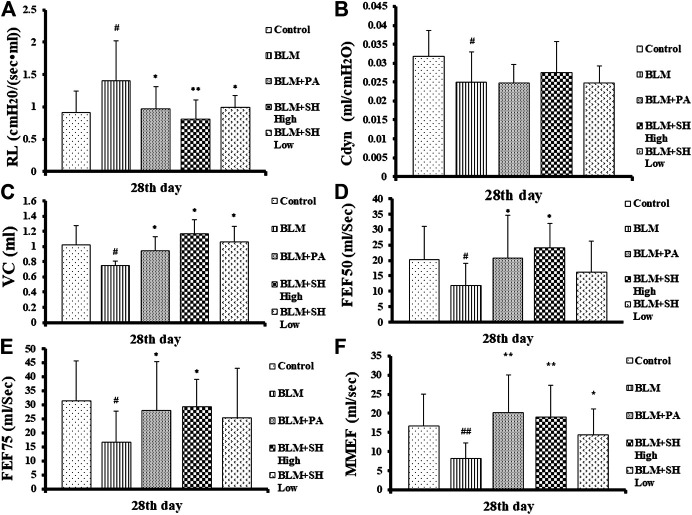
Resistance of lung (RL) **(A)**, dynamic pulmonary compliance (Cdyn) **(B)**, vital capacity (VC) **(C)** and forced expiratory flow at 50% of forced vital capacity (FEF50) **(D)**, forced expiratory flow at 75% of forced vital capacity (FEF75) **(E)**, maximum mid-expiratory flow (MMEF) **(F)** changes in BLM and SH treated mice. Each mouse was treated with saline (Control), BLM alone (BLM), BLM plus prednisone acetate (BLM + PA), BLM plus 90 mg/kg Sodium Houttuyfonate (BLM + SH High), or BLM plus 45 mg/kg Sodium Houttuyfonate (BLM + SH Low) separately. Each bar represents the mean ± SD in each group (*n* = 6–10, the number of mice sacrificed in each group was 6, 8 and 10 on Day 7, 14, and 28 respectively). #*p* < 0.05, ##*p* < 0.01 compared with Control group, ∗*p* < 0.05, ∗∗*p* < 0.01 compared with BLM group, in the same time course.

Organic lesions often lead to organ dysfunction, PF patients often accompanied with lung dysfunction. As shown in Figure 1, the indexes of VC, FEF50, FEF75 and MMFE elevated in PA and SH groups (both high and low concentration), and the index of RL decreased, with significant statistical differences compared with that in BLM mice (*p < 0.05, **p < 0.01).

### Effects of SH on Lung/Body Weight Ratio in BLM Mice

**FIGURE 2:**
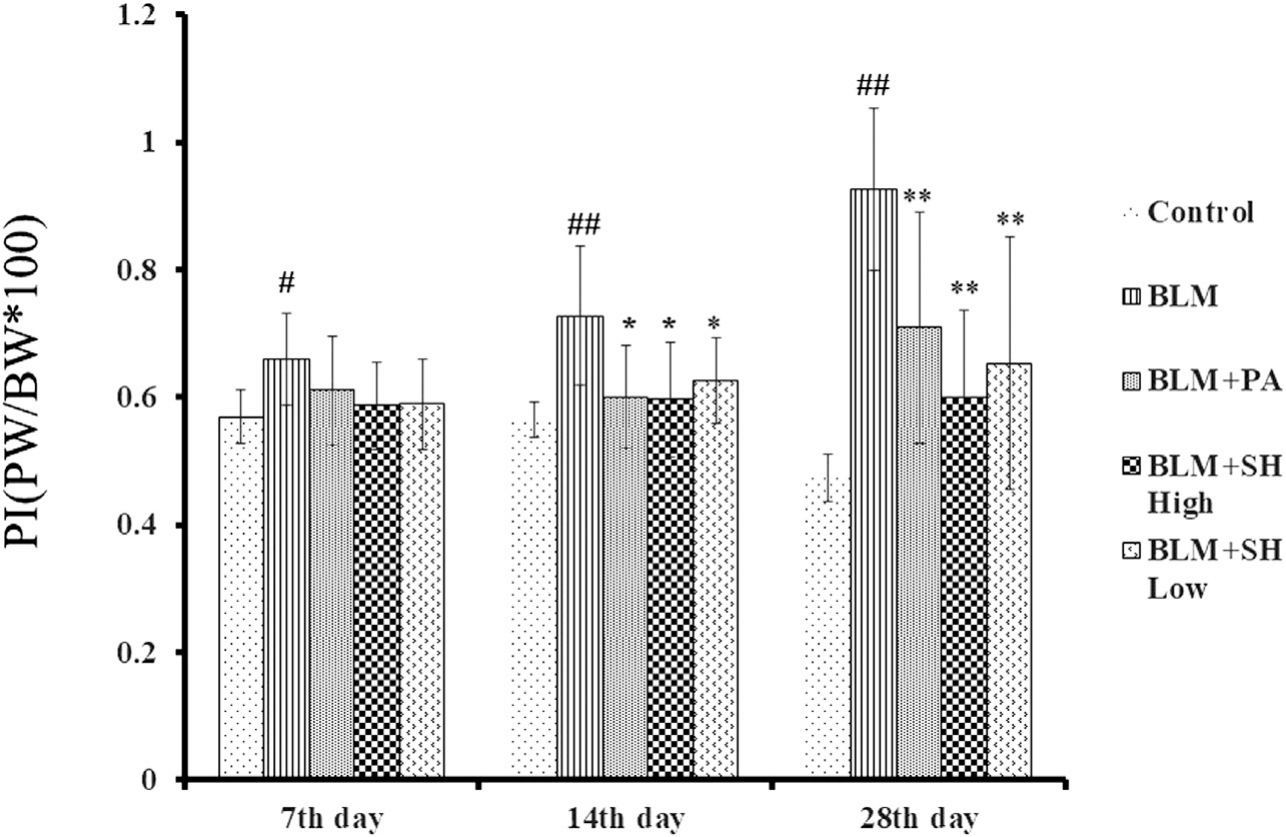
Effect of SH on lung/body weight ratio in BLM mice. Each bar represents the mean ± SD in each group (*n* = 6–10). #*p* < 0.05, ##*p* < 0.01 compared with Control group, ∗*p* < 0.05, ∗∗*p* < 0.01 compared with BLM group, in the same time course.

After instillation of saline or BLM, the lung/body weight ratio was measured on Day 7, 14 and 28. As shown in Figure 2, the lung/body weight ratio of the mice increased significantly on Day 14 and 28 after BLM treatment, which suggested that the PF mouse model was successfully developed. Both PA and SH significantly reduced the lung/body weight ratio in the Day 14 and 28 BLM-treated mice (*p < 0.05, **p < 0.01). There was no significant difference among all groups in body weight (Supplementary Figure S2).

### Effects of SH on Histological Changes in BLM Mice

**FIGURE 3:**
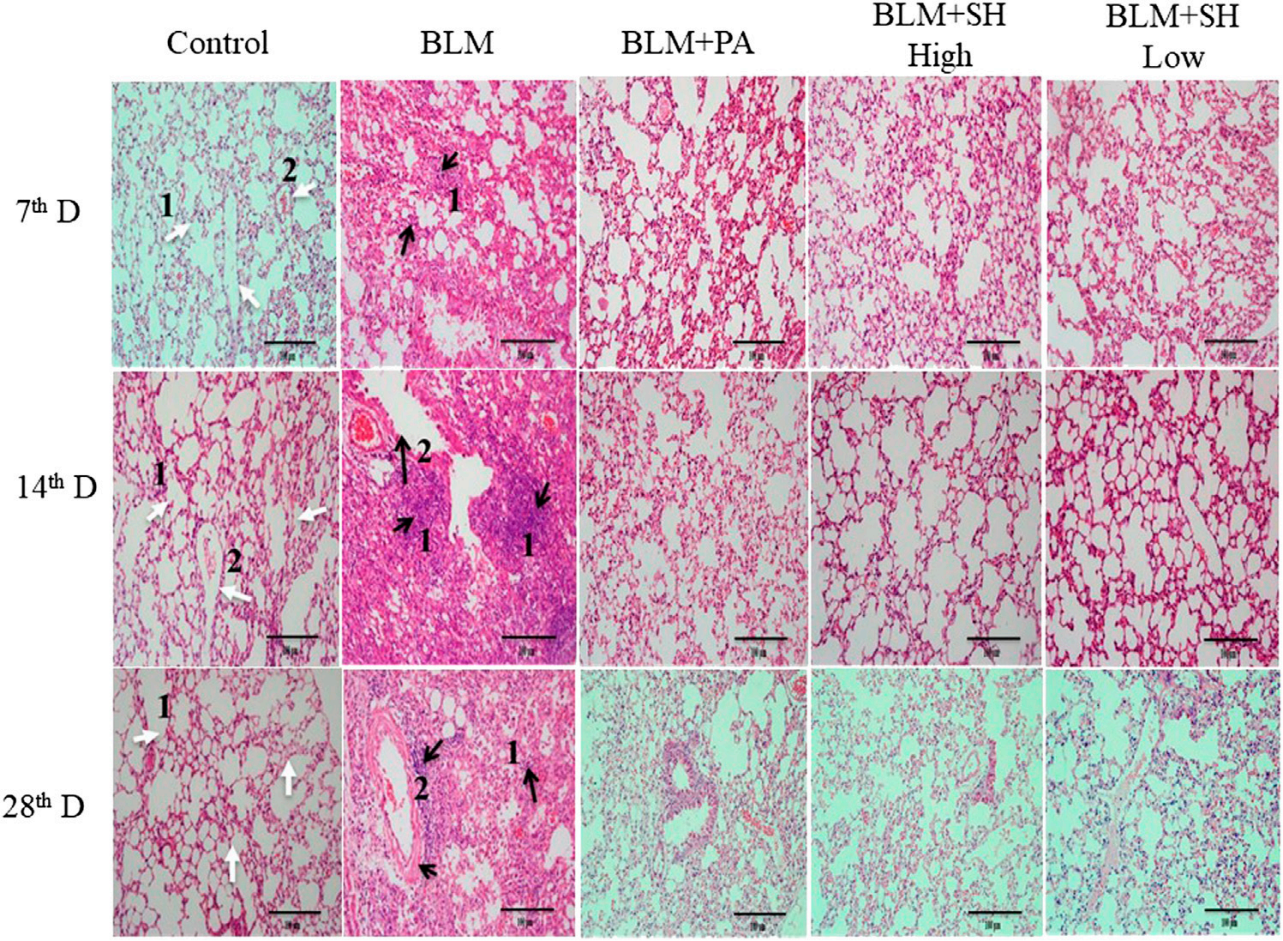
Lung tissue morphological changes in BLM and SH treated mice (H and E staining, ×200 magnification), scale bar = 100 µm. From left to right: Control group (Control); Bleomycin treated group (BLM); Bleomycin and prednisone acetate treated group (BLM + PA); Bleomycin and sodium houttuyfonate 90 mg/kg treatment group (BLM + SH High); Bleomycin and sodium houttuyfonate 45 mg/kg treatment group (BLM + SH Low). From Top to bottom: Samples were collected at seventh, 14th and 28th day after BLM administration. The inflammatory cells and fibrosis tissues were marked by black arrows; normal tissues were marked by white arrows. White arrow one indicates normal alveolar space; white arrow two indicates normal pulmonary vein. The black arrow one indicates infiltration by inflammatory cells; the black arrow two indicates the degeneration and necrosis of the bronchiole wall in the lung.

Histological examinations were performed to investigate the effects of SH on lung morphology in BLM mice. As shown in Figure 3, BLM markedly increased lung inflammatory cell infiltration and edema in H and E stained slides in a time dependent manner (Figure 3 Control and BLM panels). Thickening of the alveolar wall, infiltration of inflammatory cells and fibrosis of alveolar septa were also observed in BLM-treated mice. Fibrotic lesions clearly developed in the interalveolar area of mouse lung 14 days after bleomycin treatment and progressed to Day 28. The PA and SH treatment (both high and low concentration) reduced the lung inflammatory cell infiltration and collagen deposition (Figure 3 BLM + PA, BLM + SH High, BLM + SH Low panels).

### Effects of SH on Collagen Deposition in BLM Mice

**FIGURE 4:**
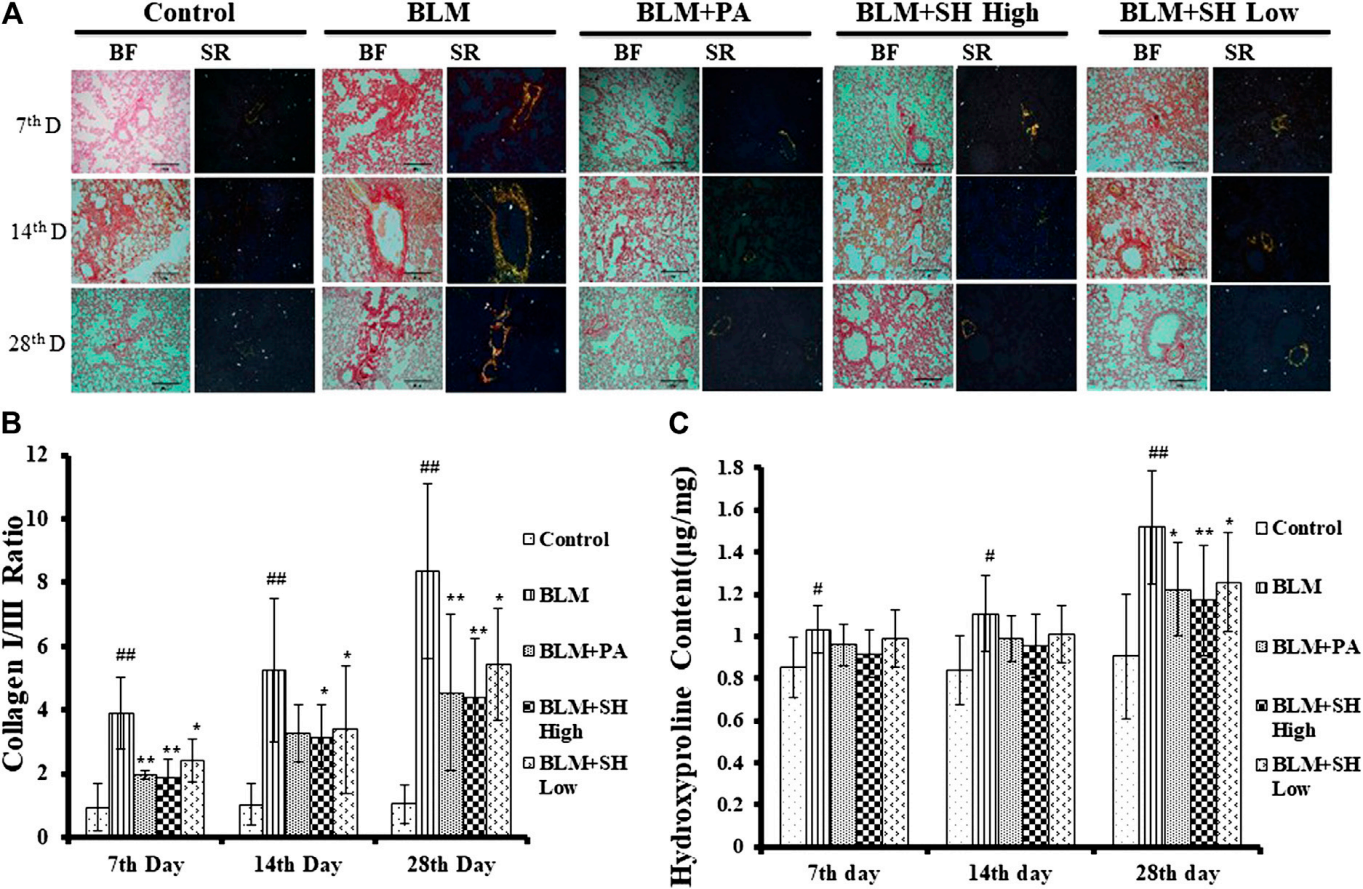
SH attenuated BLM-induced lung fibrosis. Lungs were removed from the animals on the seventh, 14th and 28th day after treatment with saline (Control), BLM alone (BLM), BLM plus prednisone acetate (BLM + PA), BLM plus 90 mg/kg Sodium Houttuyfonate (BLM + SH High), or BLM plus 45 mg/kg Sodium Houttuyfonate (BLM + SH Low) as described in Materials and Methods. **(A)** Sirius Red staining of each group in different time course. Images were adapted in Bright Field (BF) and Polarized light (Sirius Red, SR) in the same field (×200 magnification), scale bar = 100 µm. **(B)** Collagen I and III Ratio in each group in different time course. Ratio was calculated by measuring the intensity of Orange/yellow color (Collagen I) and green color (Collagen III). Samples were from more than three animals and statistic results were from more than five images in each sample. **(C)** Hydroxyproline contents in each group in different time course. Each bar represents the mean ± SD in each group (*n* = 6–10). #*p* < 0.05, ##*p* < 0.01 compared with Control group, ∗*p* < 0.05, ∗∗*p* < 0.01 compared with BLM group, in the same time course.

The key feature of fibrosis is the excessive deposition of extracellular matrix (ECM), such as collagen (Pardo and Selman, 2006; Crestani et al., 2007). Sirius Red staining was employed to examine the pathological changes of collagens. In our experiments, sections of five μm-thick were stained to identify collagen fibers. Under polarized light observation, increased amounts of type III collagen (green) and type I collagen (yellow) were observed, which were deposited in a disordered pattern in areas of fibrosis (Figure 4A, BLM group). Lung sections from control animals did not show this (Figure 4A, Control group). PA and SH treatment reduced the deposition of collagens in BLM groups in all the sample collection time points (Figure 4A, BLM + PA, BLM + SH High, BLM + SH Low group). The increase of collagen I/collagen III ratio was one of the markers for PF development (Phan et al., 1985). To investigate the change of collagen I/III ratio, we analyzed the collagen contents in various group (Figure 4B). Our data showed that BLM treatment increased the collagen I/III ratio from Day 7 to Day 28. All three drug administration groups (BLM + PA, BLM + SH High, BLM + SH Low) appeared to have low collagen I/III ratios at all-time points. The hydroxyproline content in lung tissues was used as a quantitative index of fibrogenesis and fibrosis (Martins et al., 2009). Therefore, we examined the hydroxyproline contents in the BLM and the BLM plus treatment groups (Figure 4C). Both PA and SH significantly suppressed the hydroxyproline production in the Day 28 samples (*p < 0.05, **p < 0.01). Our results suggested that SH could prevent BLM induced PF in mice through decreasing collagen production and the collagen I/III ratio. Hydroxyproline content analysis further proved that SH might have a similar effect as PA against fibrogenesis and fibrosis.

### Effects of SH on IL-1β, IL-6 and TNF-α Production in BLM Mice

**FIGURE 5:**
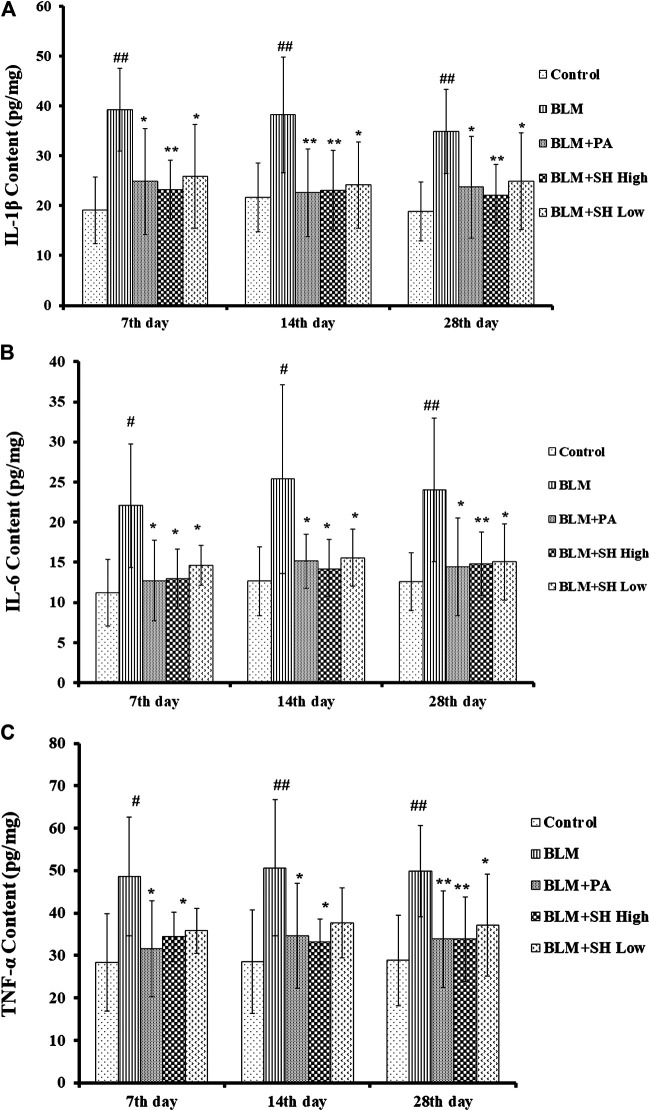
SH attenuated IL-1β, IL-6 and TNF-α protein level in BLM mice. **(A, B, and C)** showed the IL-1β, IL-6 and TNF-α protein level respectively. Lung samples were from the animals (*n* = 6–10) on the seventh, 14th and 28th day after treatment with saline (Control), BLM alone (BLM), BLM plus prednisone acetate (BLM + PA), BLM plus 90 mg/kg Sodium Houttuyfonate (BLM + SH High), or BLM plus 45 mg/kg Sodium Houttuyfonate (BLM + SH Low) as described in Materials and Methods. Each bar represents the mean ± SD in each group (*n* = 8–10). #*p* < 0.05, ##*p* < 0.01 compared with Control group, ∗*p* < 0.05, ∗∗*p* < 0.01 compared with BLM group, in the same time course.

Cytokines are the major mediators of inflammation that involved in attracting and stimulating inflammatory cells, tissue repair and fibrosis, and the downregulation of inflammatory responses (Martinet et al., 1996; Vaillant et al., 1996). It has been shown that IL-1β, IL-6 and TNF-α are the key players in acute lung injury and PF both *in vitro* and *in vivo* (Gao et al., 2011; Rhee et al., 2011). In our study, the level of IL-1β in lung homogenates prepared from the BLM-treated mice peaked on Day 7, and then decreased slightly up to Day 28 (Figure 5A). The level of IL-6 and TNF-α in the lung homogenates of the BLM-treated mice was maintained throughout the time course (Figures 5B,C). All values in the BLM group were significantly elevated compared to those in the control group. The administration of SH or PA attenuated the production of IL-1β, IL-6 and TNF-α compared to the control throughout the time course (Figures 5A–C). Interestingly, high concentration SH had greater suppressive effects than PA in some time points (Figure 5A Day 7, 28; Figure 5B Day 14; Figure 5C Day 14).

### Effect of SH on TGF-β1 mRNA Level in BLM Mice

**FIGURE 6:**
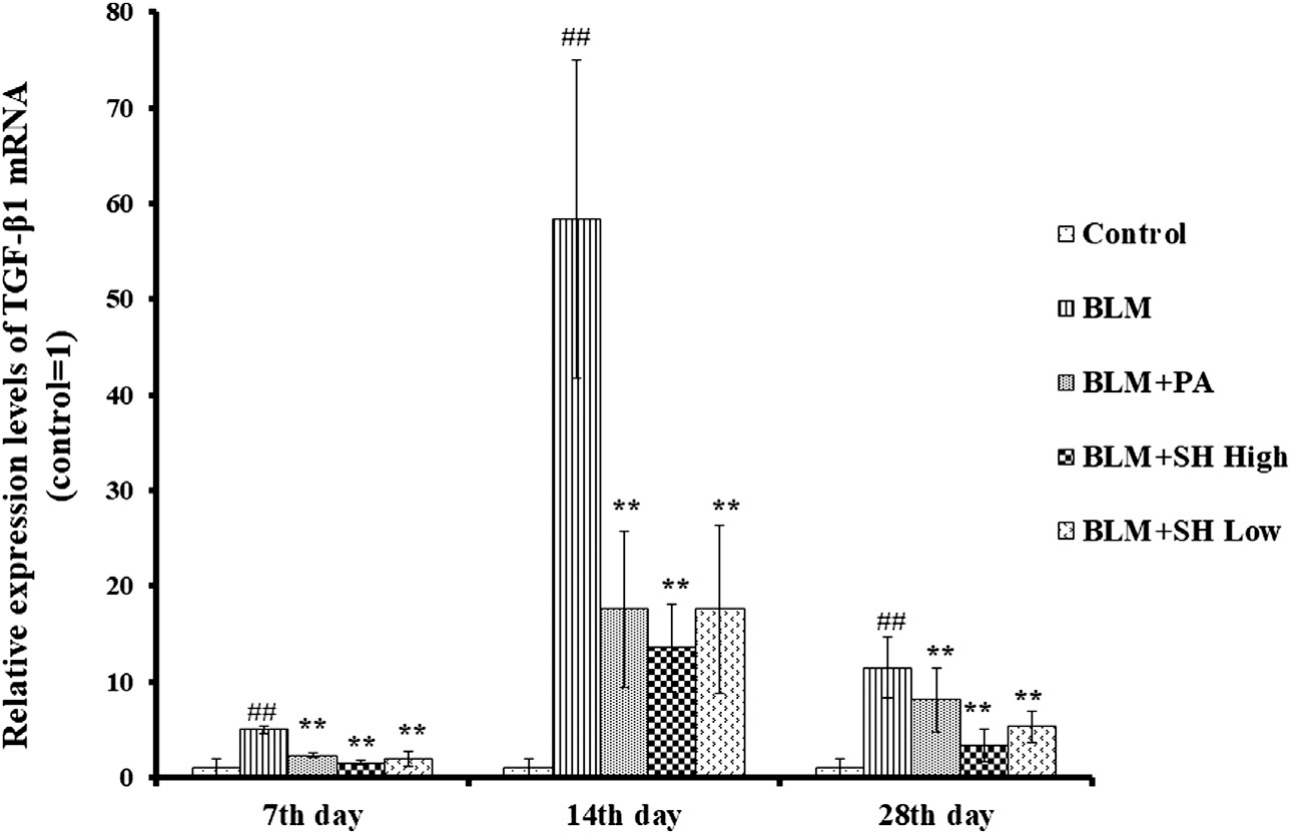
SH decreased TGF-β1 mRNA level in BLM mice. TGF-β1 mRNA from lung tissue was extracted and analyzed as described in Materials and Methods. Lung tissues were from three animals and the data showed the relative mRNA level compared to GAPDH. Each bar represents the mean ± SD in each group (*n* = 3). **#** *p* < 0.01 compared with Control group, ∗*p* < 0.01 compared with BLM group, in the same time course.

TGF-β1, as one of profibrotic cytokines, could mediate fibroblast proliferation, transformation and accumulation, leading to ECM production and deposition, tissue destruction, and lung function loss (Sime and O’Reilly, 2001). Several independent investigations have shown that inhibition of TGF-β1 is an effective means to potentially inhibit PF (Pardo and Selman, 2002; Chen and Stubbe, 2005). Then we used real time quantitative-PCR to examine the mRNA level of TGF-β1 in BLM and various drug treated mice. As shown in Figure 6, BLM increased the TGF-β1 mRNA level more than the control group at Day 7, 14 and 28. Both SH and PA attenuated the TGF-β1 mRNA elevation in all the time course.

### Effects of SH on TGF-β1 and pSmad2/3 Protein in BLM Mice

**FIGURE 7:**
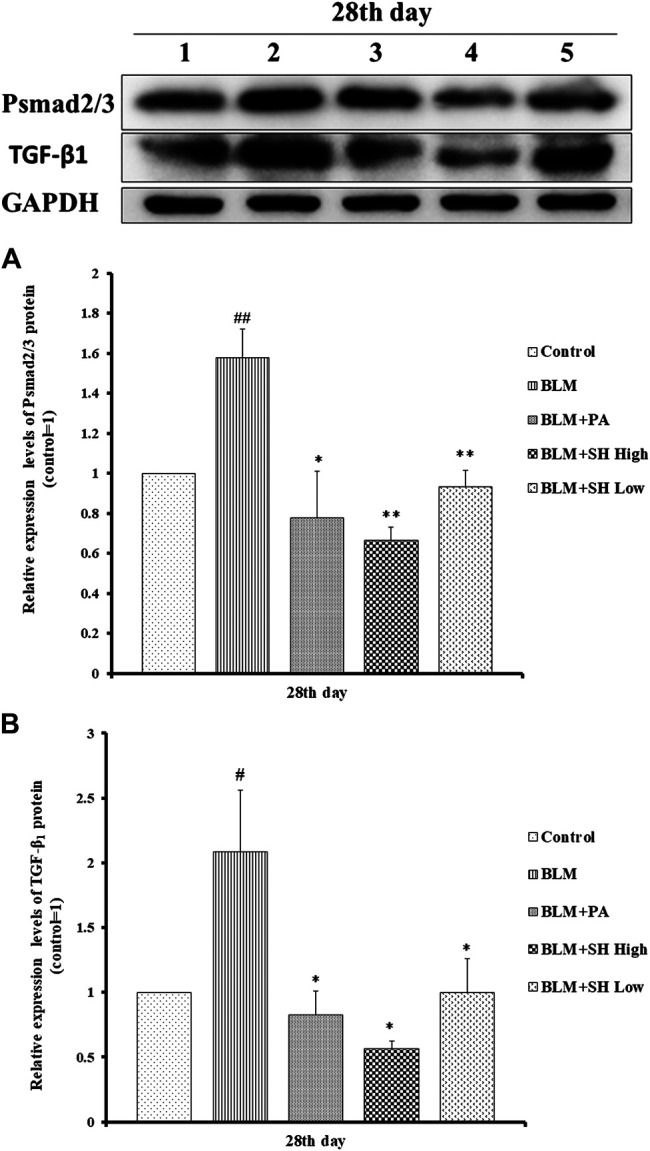
SH attenuated TGF-β1 and pSmad2/3 protein level in BLM mice. **(A and B)**, Protein sample from lung tissue was extracted and analyzed as described in Materials and Methods. One is treated with saline (Control), two is treated with BLM alone (BLM), three is treated with BLM plus prednisone acetate (BLM + PA) four is treated with BLM plus 90 mg/kg Sodium Houttuyfonate (BLM + SH High), five is treated with BLM plus 45 mg/kg Sodium Houttuyfonate (BLM + SH Low). Figure 7B Each bar represents the mean ± SD in each group (*n* = 8–10). #*p* < 0.05, ##*p* < 0.01 compared with Control group, ∗*p* < 0.05, ∗∗*p* < 0.01 compared with BLM group, in the same time course.

Changes in the expression of TGF-β1 and phosphorylated Smad2/3 proteins were detected to reflect the extent of PF (Kamato et al., 2013; Jia et al., 2019). PF will be accommodated though Smad pathway which is mediated by TGF-β1 by N-acetyl-serine-aspartate-lysyl-proline (AcSDKP). TGF-β1 and pSmad2/3 protein expressions were significantly down-regulated both in the PA and SH groups (both high and low concentration) compared with BLM mice (Figure 7).

## Discussion

PF, a chronic progressive lung disease, is characterized by abnormal wound healing with lung destruction and dysfunction (Pardo and Selman, 2002; Scotton and Chambers, 2007). Pulmonary fibrosis is a complex chronic process, in addition to fibroblast activation, involving multiple mechanisms such as alterations in the immune regulation, chronic inflammation and participation of many cells, ECM, cytokines, various regulatory enzymes, etc. (Wick et al., 2013). Besides the recognized role of stimulation pathways, the importance of natural counter-regulatory mechanisms responsible for maintaining tissue function by limiting inflammatory and fibrotic responses has also been recognized. Evidence increasingly suggests that chronic inflammation is caused by an insufficient generation of anti-inflammatory and pro-resolving mediators, while fibrosis is caused by an insufficient production of inhibitory signals that control fibroblast function (Martinet et al., 1996; Wilson and Wynn, 2009). The basic pathological feature of pulmonary interstitial fibrosis is the massive deposition of ECM and collagen is one of the components of ECM. Collage I and collagen III are the dominant collagens in pulmonary tissues. Collagen is rich in hydroxyproline and the increase of collagen I/III ratio and hydroxyproline content can reflect the deposition of collagen. In addition, successful model establishment and amendment in PF mouse model can also be reflected indirectly by mouse lung/body weight ratio variation.

In this study, we demonstrated that the administration of SH suppressed the increase in lung/body weight ratio, pulmonary collagens contents and hydroxyproline contents in mice induced by BLM, and lung functions also been improved. To examine and confirm the protective properties of SH, we designed two SH treatment groups: the high concentration (90 mg/kg) and low concentration (45 mg/kg) groups. These results indicate that SH has the potential to reduce BLM toxicity, particularly PF, and can be a candidate drug for IPF. BLM is widely used in animal models of IPF as a typical inducer of PF (Moeller et al., 2008; Mouratis and Aidinis, 2011). BLM has also been clinically used for standard cancer therapies such as squamous cell carcinomas and lymphomas (Carver et al., 2007). However, due to the risk of severe pulmonary toxicity, its clinical use is restricted. So, how to prevent this adverse reaction remains unsolved (Chen and Stubbe, 2005). The results of our study may help solve this problem by preventing pulmonary toxicity in patients administered with BLM.

The role of SH in the regulation of the inflammatory response is of particular interest as the compound not only inhibits pro-inflammatory factors but actively participates in the resolution of inflammation, inhibiting an over-exuberant inflammatory response and restricting injury to the host. The etiology of PF is mainly manifested by inflammatory and fibro-proliferative changes through the release of inflammatory cytokines such as TNF-α, IL-1β, IL-4, IL-13, IFN-γ and TGF-β, etc. (Kandhare et al., 2015). With its significances such as in mimicking the chronic aspect of pulmonary fibrosis and releasing of inflammatory cytokines, BLM is widely used in rodent models. After bleomycin administration, mice developed acute lung injury with macrophage and neutrophil infiltration into the tissues. And it was demonstrated that macrophages accumulating in the lung at early were activated toward a proinflammatory M1 phenotype, releasing proteolytic enzymes, ROS, proinflammatory cytokines including IL-1, IL-6, IL-18, TNF-α, chemokines, etc. This was followed by the increase of anti-inflammatory/wound repair M2 macrophages, releasing IL-4, IL-13, etc. (Laskin et al., 2019). SH showed the ability to inhibit cytokine (IL-1β, IL-6 and TNF-α) production in lung tissues of BLM mice, suggesting that SH could also be used as an anti-inflammatory drug in lung infections. Indeed, SH is widely used as an antivirus agent in clinical practice. Despite its long clinical use as an anti-inflammatory drug, this study is the first to describe the value of SH on cytokine expression in BLM mice and has provided further experimental evidences on how SH exerts positive effects on PF in mice. Previous reports have shown that in a chronic obstructive pulmonary disease (COPD) inflammatory model, SH attenuated inflammation by reducing the mRNA levels of TLR4, MyD88, and NF-κ B p65 and the protein expression of TLR4 and p65. SH also attenuated intestinal inflammation through the NF-κB pathway by inhibiting the expression of *p*-IκBα and p-p65 (Wu et al., 2017; Zhang et al., 2020). However, the mechanism of SH inhibiting the increase of inflammatory cytokines in BLM-treated mice has not yet been reported and whether the NF-κB pathway has played certain role requires further study.

The key feature of fibrosis, namely the excessive deposition of ECM such as collagen, was markedly inhibited by SH in BLM mice. The presence of myofibroblasts in lung tissues was proved both in PF patients and animal models (Phan et al., 1985). TGF-β1 is the primary cytokine related to PF within many mediators, which is involved in the transformation of fibroblasts into myofibroblasts and the induction of epithelial-mesenchymal transformation (EMT) in tissue fibrosis and synthesis of collagen, etc. (Caraci et al., 2008; Yamazaki et al., 2017). Smad proteins also have been proved to be involved in signal transducers for TGF-β1 signaling pathway where phosphorylated Smad2/3 proteins translocated into the nucleus upon binding with Smad4 to regulate the transcription of target genes and participate in PF (Attisano and Wrana, 2002). This study showed that SH significantly suppressed the mRNA level and the protein expression of TGF-β1 and pSmad2/3 in BLM mice, implying that SH, through down-regulation of TGFβ1-Smad2/3 signal transduction, could at least partly alleviate BLM-induced formation and proliferation of myofibroblast.

## Conclusion

In summary, we demonstrated the beneficial effects of SH in the prevention of PF induced by BLM in mice. Our results suggest that this compound is an attractive candidate against IPF and pulmonary toxicity induced by BLM.

## Data Availability

The raw data supporting the conclusion of this article will be made available by the authors, without undue reservation, to any qualified researcher.
