# Supplementary material for: Sodium Houttuyfonate Inhibits Bleomycin Induced Pulmonary Fibrosis in Mice
Source: Front Pharmacol. 2021 Feb 25;12:596492. doi: 10.3389/fphar.2021.596492 (PMC7947865; doi:10.3389/fphar.2021.596492)
Supplement: Supplementary file 1 [file presentation1.pptx]

## Slide 1
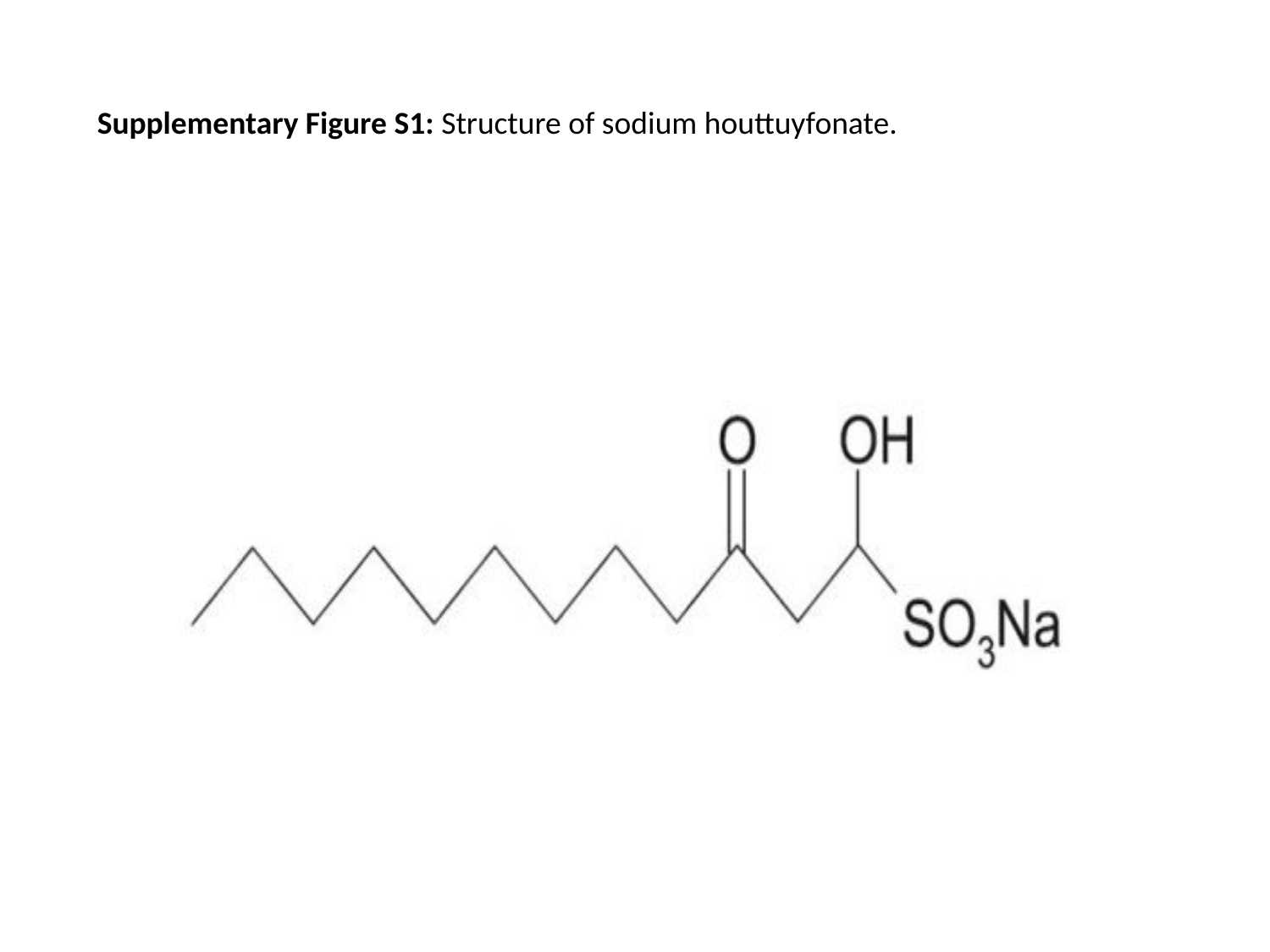

Supplementary Figure S1: Structure of sodium houttuyfonate.

## Slide 2
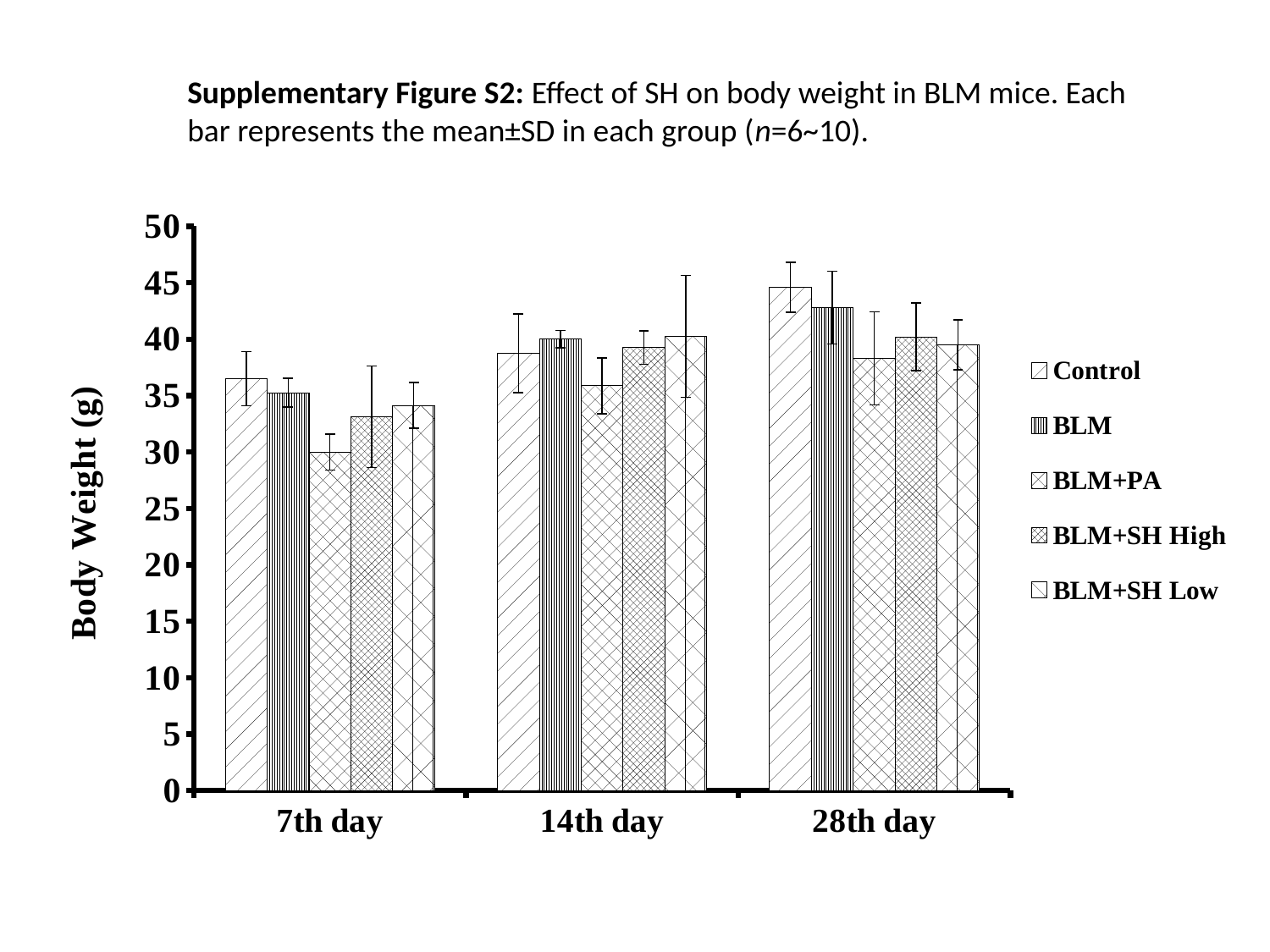

Supplementary Figure S2: Effect of SH on body weight in BLM mice. Each bar represents the mean±SD in each group (n=6~10).
### Chart
| Category | | | | | |
|---|---|---|---|---|---|
| 7th day | 36.5 | 35.25 | 30.0 | 33.125 | 34.125 |
| 14th day | 38.75 | 40.0 | 35.875 | 39.25 | 40.25 |
| 28th day | 44.6 | 42.8 | 38.3 | 40.2 | 39.5 |

## Slide 3
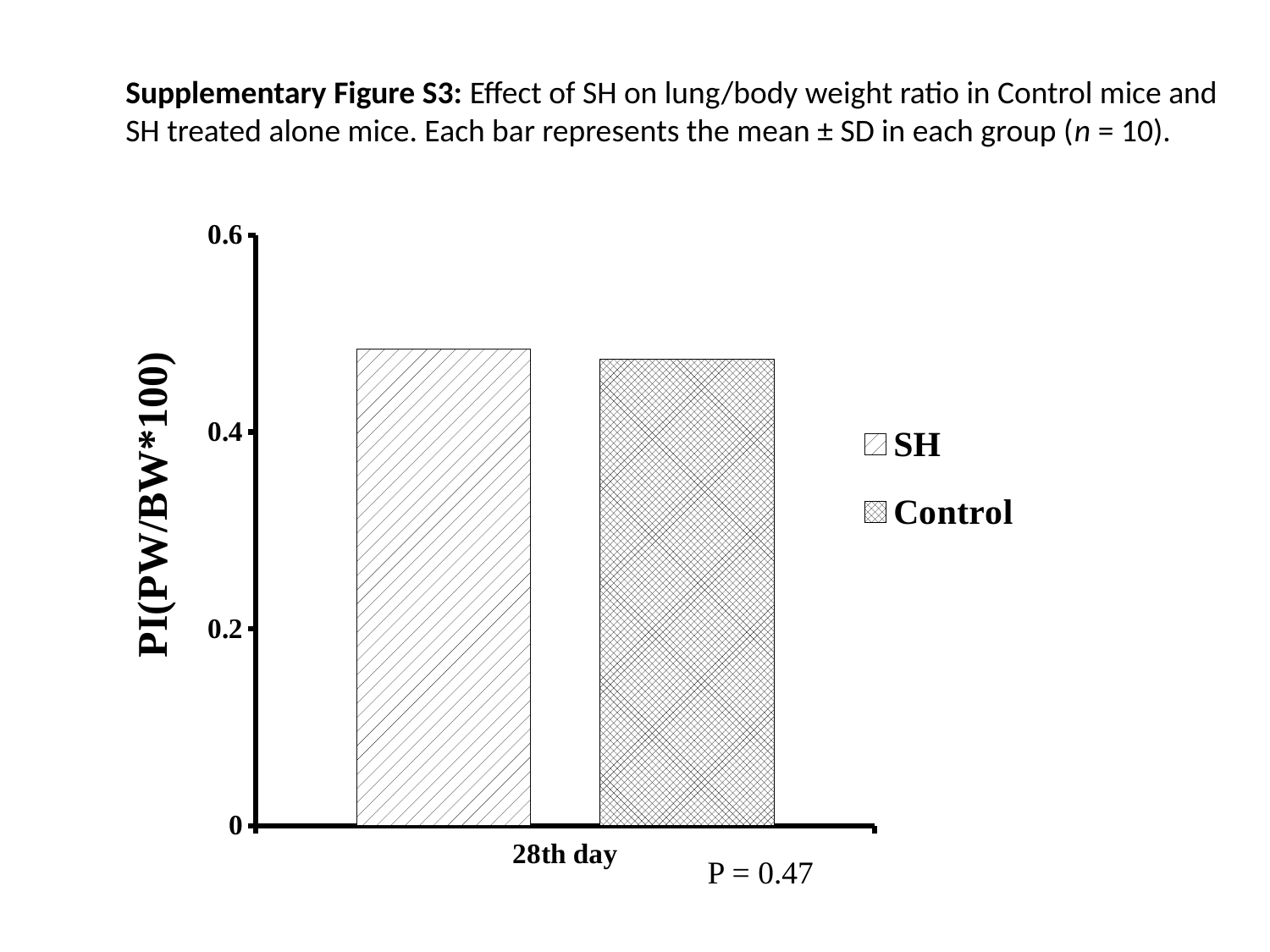

Supplementary Figure S3: Effect of SH on lung/body weight ratio in Control mice and SH treated alone mice. Each bar represents the mean ± SD in each group (n = 10).
### Chart
| Category | SH | Control |
|---|---|---|
| 28th day | 0.48400830997842803 | 0.4738886941316343 |P = 0.47

## Slide 4
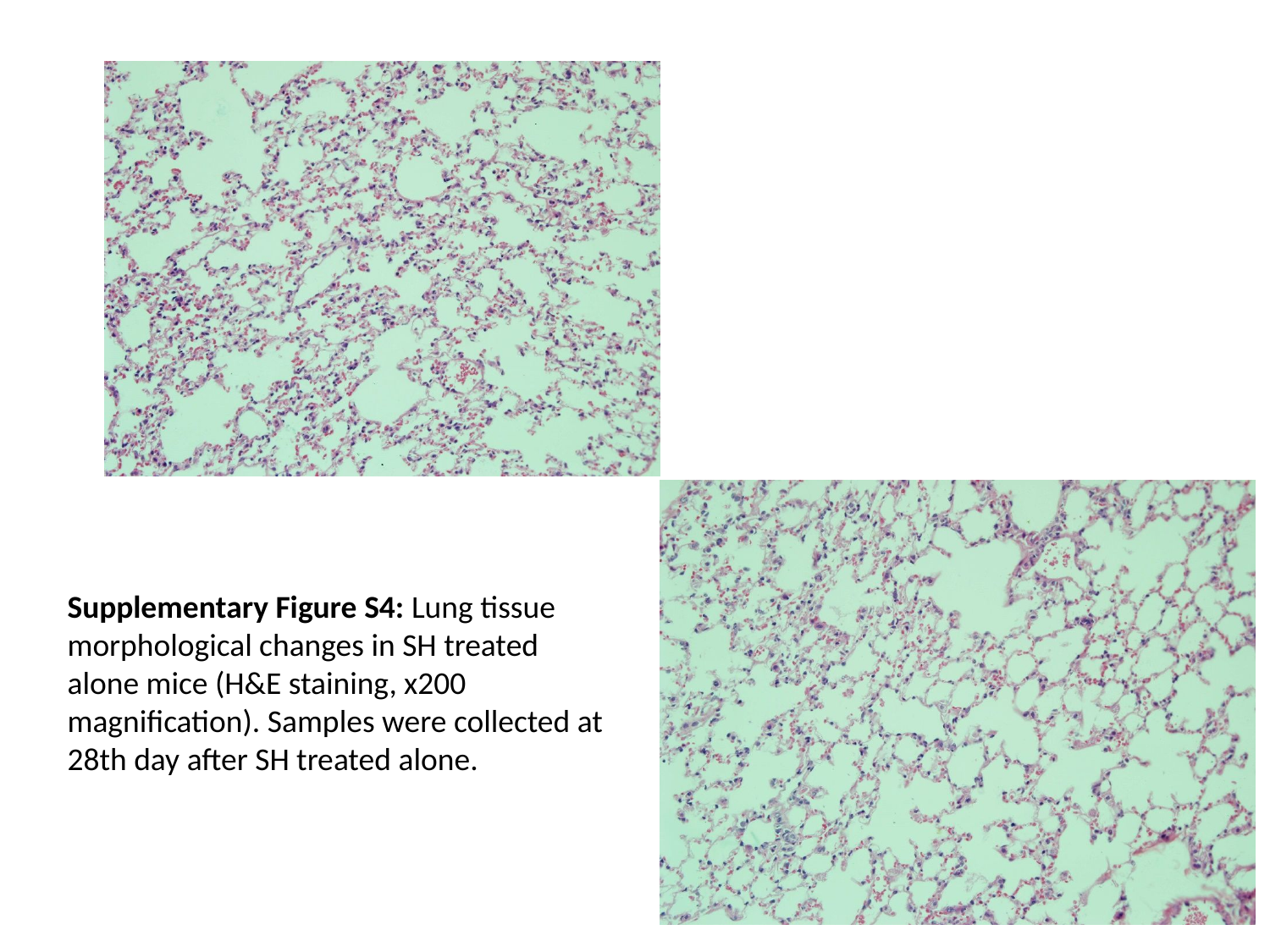

Supplementary Figure S4: Lung tissue morphological changes in SH treated alone mice (H&E staining, x200 magnification). Samples were collected at 28th day after SH treated alone.

## Slide 5
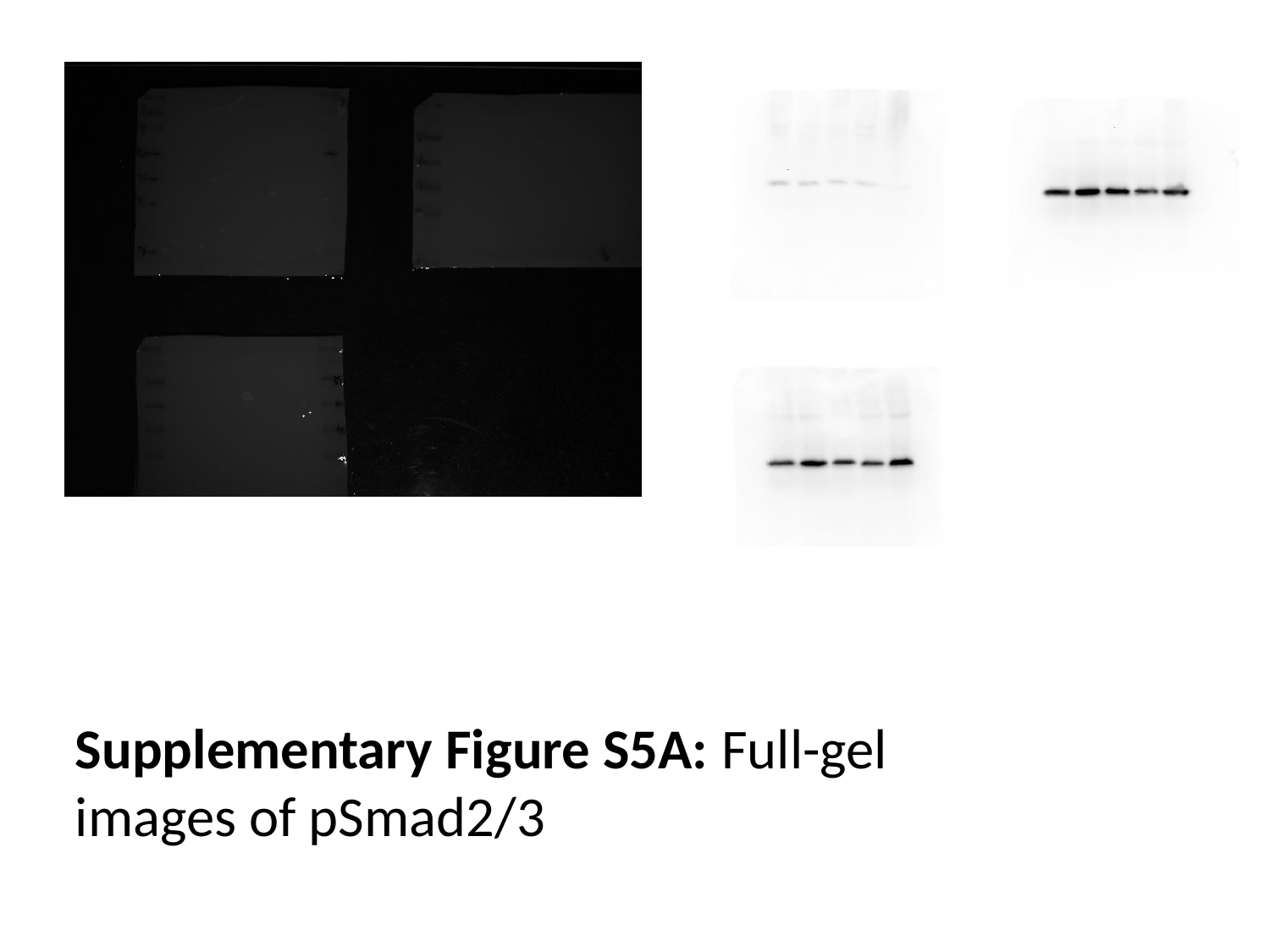

Supplementary Figure S5A: Full-gel images of pSmad2/3

## Slide 6
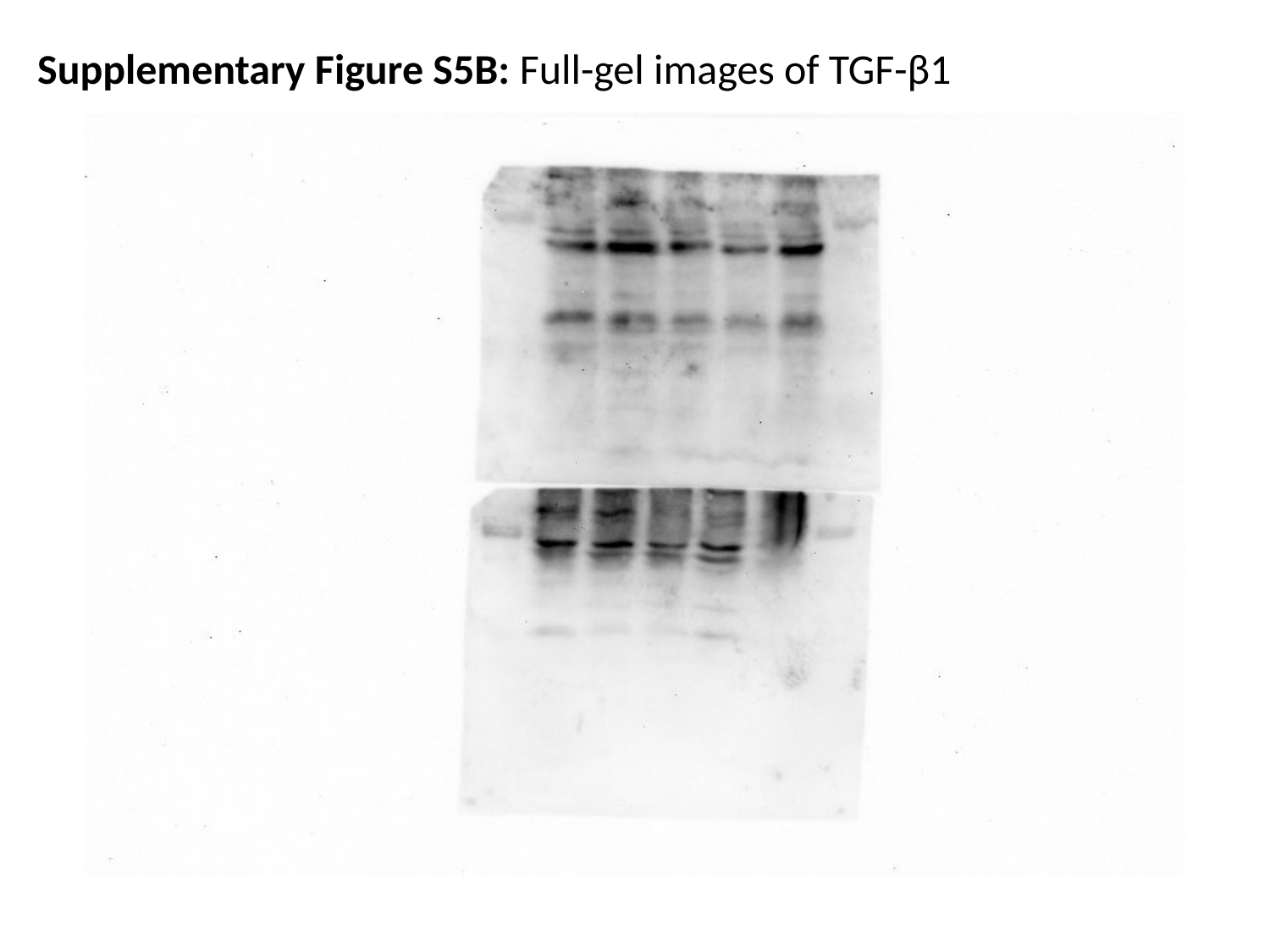

Supplementary Figure S5B: Full-gel images of TGF-β1

## Slide 7
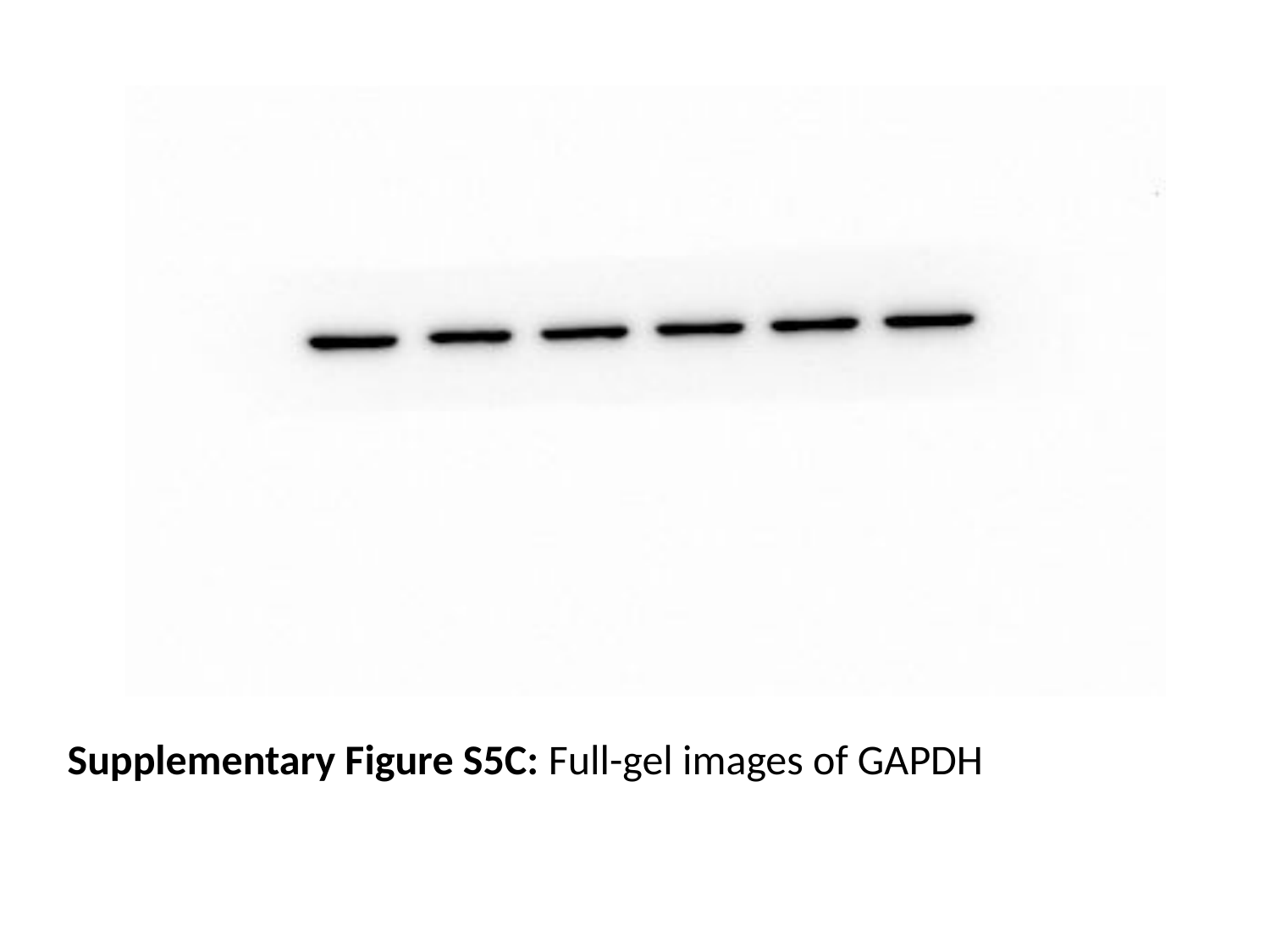

Supplementary Figure S5C: Full-gel images of GAPDH

## Slide 8
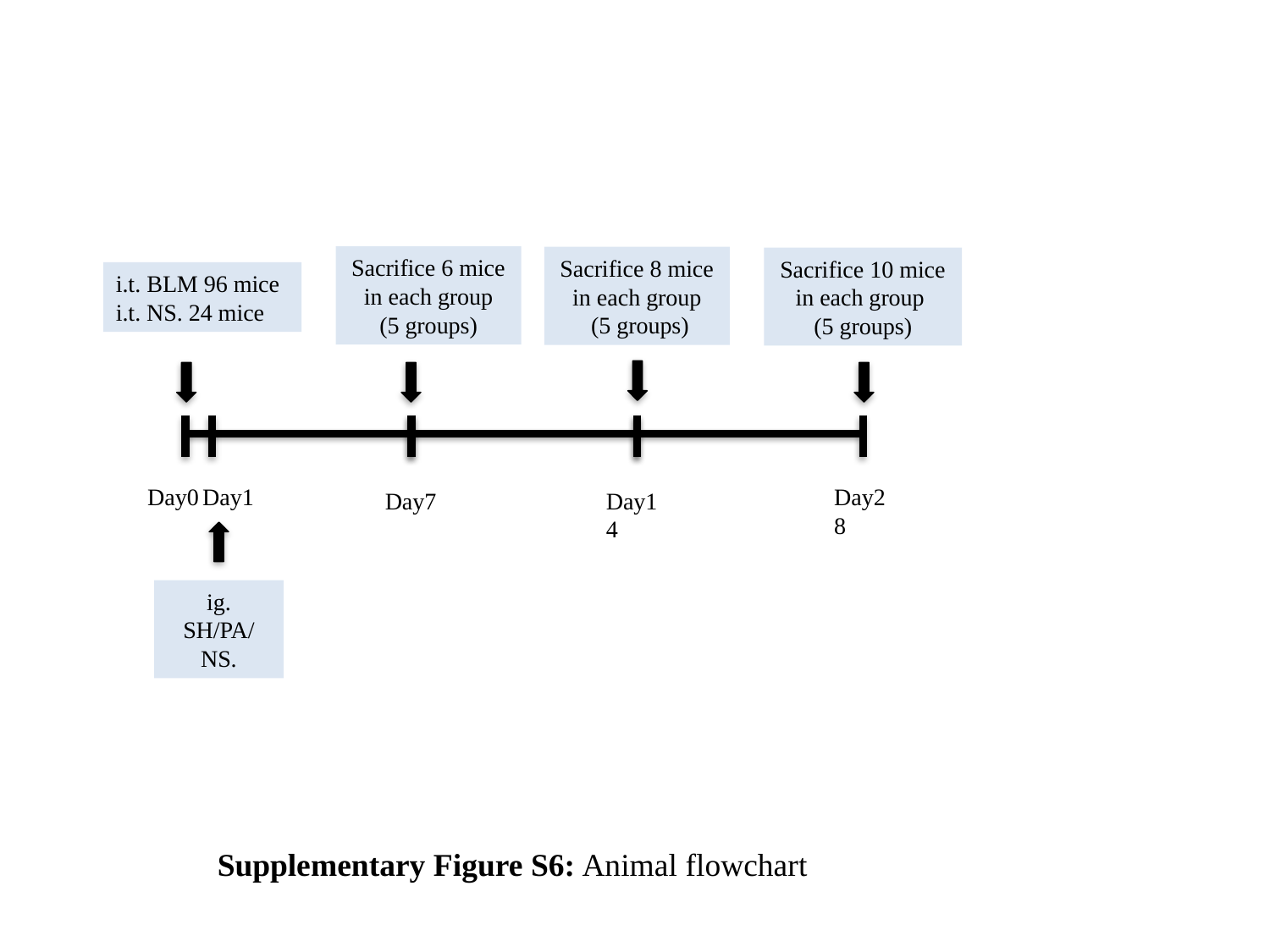

Sacrifice 6 mice in each group
(5 groups)
Sacrifice 8 mice in each group
 (5 groups)
Sacrifice 10 mice in each group
(5 groups)
i.t. BLM 96 mice
i.t. NS. 24 mice
Day0
Day1
Day28
Day7
Day14
ig.
SH/PA/NS.
Supplementary Figure S6: Animal flowchart
